# Understanding the impact of soil microbiome on strawberry growth and nutritional profiles

**DOI:** 10.3389/fmicb.2025.1654776

**Published:** 2025-09-09

**Authors:** Min-Jin Kwak, Sriniwas Pandey, Yuting Zhai, Bugil Choi, Thanyachanok Sutthanonkul, Won Suk Lee, Kwangcheol C. Jeong

**Affiliations:** ^1^Emerging Pathogens Institute, University of Florida, Gainesville, FL, United States; ^2^Department of Animal Sciences, University of Florida, Gainesville, FL, United States; ^3^School of Plant, Environmental and Soil Sciences, Louisiana State University, Baton Rouge, LA, United States; ^4^Department of Agricultural and Biological Engineering, University of Florida, Gainesville, FL, United States

**Keywords:** rhizosphere microbiome, strawberry, nutrient uptake, food security, food safety

## Abstract

The rhizosphere microbiome plays an important role in plant growth, nutrient acquisition, and overall health. In this study, we investigated the relationship between the rhizosphere microbiome and the health status of strawberries (*Fragaria* × *ananassa*) under identical soil and environmental conditions. Strawberry plants were categorized into a healthy group (H) and an unhealthy group (UH) based on morphological characteristics, and the soil microbial community was analyzed using 16S rRNA gene sequencing. The H group exhibited significantly higher nitrogen concentrations, whereas the UH group showed excessive accumulation of iron, manganese, zinc, and copper. Microbiota analysis revealed distinct structural differences between the H and UH groups, with several bacterial taxa displaying significant differences in relative abundance. Notably, *Microvirga* and JG30-KF-CM45 emerged as key bacterial taxa associated with plant nutrient status. *Microvirga* was positively correlated with nitrogen levels but negatively associated with micronutrient accumulation, while JG30-KF-CM45 showed the opposite trend. Furthermore, co-occurrence network analysis indicated that microbial communities in the UH group were characterized by intensified competitive interactions, which may contribute to rhizosphere microbiome destabilization and impaired plant growth. These findings indicate that microbial interactions within the rhizosphere influence nutrient homeostasis and plant health. The observed microbial imbalances in UH plants suggest the importance of maintaining a stable microbial community for improved crop productivity. This study provides valuable insights into the role of rhizosphere microbiome in sustainable strawberry cultivation and underscores the potential of microbiome-based strategies to improve plant health and productivity.

## 1 Introduction

The soil microbiome particularly in the rhizosphere plays a fundamental role in plant growth, health, and productivity (Schnitzer et al., 2011). The rhizosphere is a dynamic area where plants interact with diverse microbial communities, including bacteria, fungi, archaea, and viruses, which influence nutrient cycling, pathogen suppression, and plant stress resistance (Wagg et al., 2011; Michl et al., 2023). These microbial communities can facilitate the exchange of essential nutrients, such as sodium, phosphorus, and potassium, between the soil and plant. In addition, they produce plant growth regulators, including auxins and cytokinins (Lazcano et al., 2021; Compant et al., 2025). Understanding the microbial composition and activities in the rhizosphere is therefore crucial for assessing plant performance under both optimal and stressful environmental conditions.

Strawberries (*Fragaria* × *ananassa*) are highly sensitive to soil quality and environmental stressors (Busby et al., 2017), both of which are influenced by the composition and diversity of the rhizosphere microbiome, thereby playing a critical role in modulating plant health and productivity (Yan et al., 2017). Specifically, plant growth-promoting rhizobacteria (PGPR) contribute to enhanced nutrient uptake, regulation of growth hormone production, and protection against pathogens (Yang et al., 2020). Disruptions in rhizosphere microbiome or dysbiosis can compromise plant defense mechanisms, leading to impaired growth, reduced yield, irregular plant maturation, and increased susceptibility to diseases (Siegieda et al., 2024). Consequently, the establishment and maintenance of a healthy microbiome are essential for ensuring sustainable strawberry productivity.

Certain bacterial taxa have been directly associated with improved nutrient assimilation and enhanced plant resistance to antibiotics and pathogens (Deng et al., 2019). Notably, *Pseudomonas*, *Bosea*, *Microvirga*, and *Paenibacillus* contribute to nutrient cycling and plant-microbe interactions (Xu et al., 2015; Marin et al., 2024; Wu et al., 2025). Additionally, studies have shown that plants grown in identical soil under the same environmental conditions exhibit varying growth patterns. This observation suggests that differences in the rhizosphere microbiome may play a crucial role in influencing nutrient uptakes and plant development. Therefore, we hypothesized that variations in rhizosphere microbial communities directly impact plant growth and nutrient acquisition. In this study, we cultivated strawberry plants in identical soil under uniform management conditions to investigate the relationship between the rhizosphere microbiome and plant growth performance. Furthermore, we compared plants grown differently to elucidate the role of key microbial taxa in plant health and nutrient levels. Understanding these microbial interactions is critical for enhancing crop yield, improving food safety and security by promoting healthier plant growth.

## 2 Materials and methods

### 2.1 Experimental design and sampling

**Figure 1:**
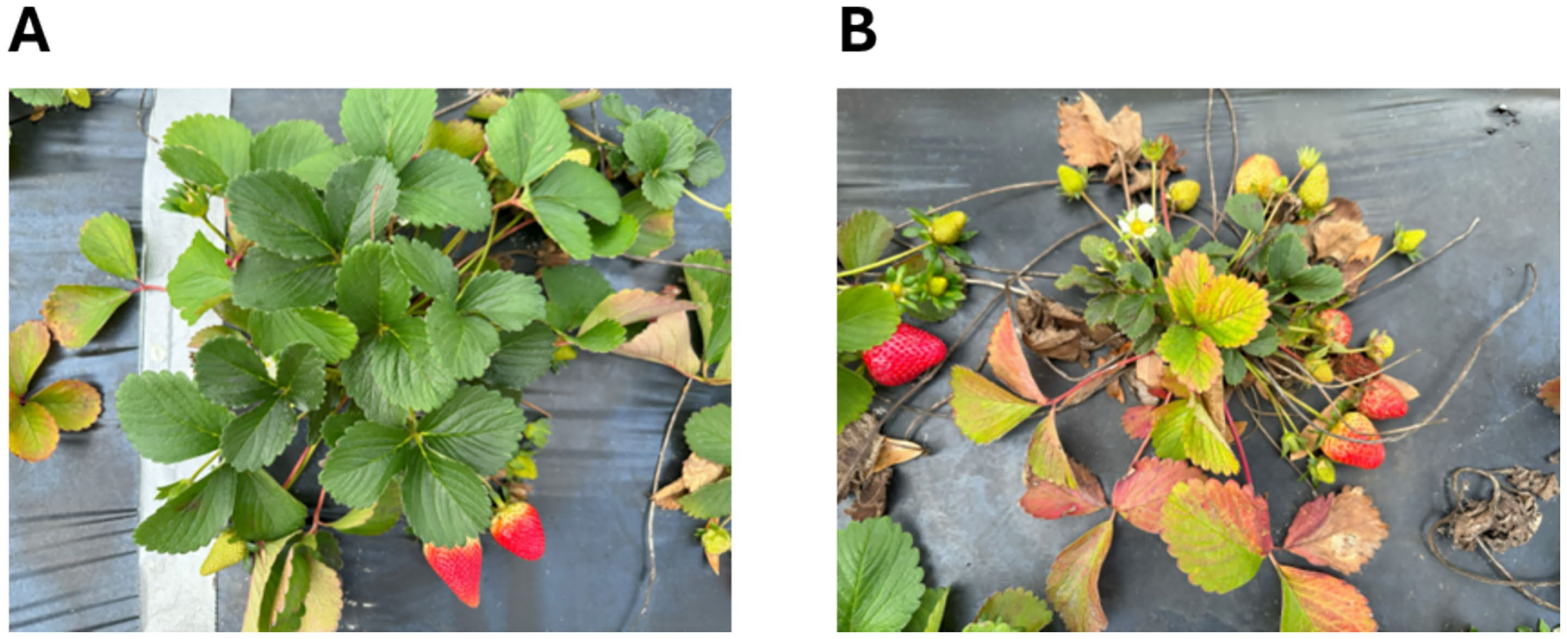
Representative images of healthy **(A)** and unhealthy **(B)** strawberry plants growing in close proximity.

Two strawberry cultivars, Brilliance and Medallion, were used in this study. They were chosen since they were most commonly grown by strawberry growers in Florida. They were planted in an experimental field located in the Plant Science Research and Education Unit (PSREU) at the University of Florida in Citra, Florida, USA on October 13th, 2023, using bare-root transplants grown in a nursery in California, USA. Standard fertilization and pest control practices were followed, with no microbial inoculants applied. The strawberries were grown in 10 raised beds, and two rows of strawberry plants were planted in each bed. The length of the bed was 61 m, and the width of each bed was 0.5 m. The plants in each row were 0.3 m apart. To minimize potential microenvironmental variability, healthy (H) and unhealthy (UH) plants were selected from adjacent locations within the same raised bed. From the field, a total of 10 healthy and 10 unhealthy plants were randomly chosen for collecting leaf and soil samples in March 2024. Most of the U and UH sample plants were grown nearby, exhibiting an interesting situation of growing status. Each group consisted of five Brilliance and five Medallion strawberries. The H plants were selected based on their bigger canopy sizes and dark green leaf conditions without any vivid signs of nutrient deficiencies, whereas the selected UH plants showed smaller canopy sizes and signs of nutrient deficiencies. Figure 1 shows an example of H and UH plants growing nearby.

Several leaves of each plant were collected and put in a sterile plastic bag for later nutrient analyses. Approximately 5 g of soil samples were collected under each plant from the root area using a cotton swab directly from root surfaces (within ~2 mm) and put in a clean plastic bag for lab analyses. The soil type was Arredondo sand with 0 to 5 percent slopes (United States Department of Agriculture, 2025).

### 2.2 Nutrient profiling of leaves

The sampled leaves were dried at 70 °C for 48 h in an oven, sieved using a 250 μm sieve, and analyzed at the Extension Soil Testing Laboratory at the University of Florida, Gainesville, Florida, USA. Leaf tissue samples were analyzed by the total Kjeldahl nitrogen (TKN) for nitrogen contents and the inductively coupled plasma (ICP) metals procedure for other elements (Mylavarapu et al., 2024).

### 2.3 16S rRNA gene sequencing and bioinformatic analysis

Genomic DNA was extracted from 0.25 g of soil based on the protocols set by the manufacturer from QIAamp DNeasy PowerSoil Pro kit (Qiagen, United States). To study the bacterial diversity, the DNA library was prepared and sequenced as described by Fan et al. (2021) and Kwak et al. (2021). Briefly, the V4 region of the 16S rRNA gene was amplified by polymerase chain reaction (PCR) with dual-index primers with the following reaction mixture: 1 μL forward index primer (10 mM), 1 μL reverse index primer (10 mM), 1 μL 10 ng/μL DNA samples, and 17 μL Pfx AccuPrime master mix (Invitrogen, United States). The PCR conditions were denaturation for 5 min at 95 °C, followed by 30 cycles of 95 °C for 30 s, annealing at 55 °C for 30 s, and extension at 72 °C. The amplicons were then subsequently purified and normalized using the SequalPrep Plate Normalization Kit (Invitrogen, United States). The DNA library was then constructed by pooling the same amount of barcoded V4 amplicons from all 20 samples. The final DNA library was loaded into MiSeq v2, 2 × 250 cycle cartridge (Illumina, United States), and sequenced using the Illumina MiSeq platform.

Raw sequencing data were acquired from the Illumina BaseSpace platform and processed using the Quantitative Insights into Microbial Ecology (QIIME 2) software (version 2024.10.). Paired-end reads were imported, and the quality of the initial bases was assessed according to the Interactive Quality Plot. The sequence quality control was conducted with the Divisive Amplicon Denoising Algorithm (DADA2) pipeline implemented in QIIME 2, including steps for filtering low-quality reads, denoising reads, merging the paired-end reads, and removing chimeric reads. The phylogenetic tree was generated using the align-to-tree-mafft-fasttree pipeline from the q2-phylogeny plugin of QIIME 2. The sequencing depth was normalized to 8,190 sequences per sample. The Shannon index and Weighted UniFrac distance were calculated using the core-metrics-phylogenetic pipeline. All amplicon sequence variants were classified into the bacterial taxonomy using the q2-feature-classifier plugin of QIIME 2 and the SILVA v138 database (Quast et al., 2012).

### 2.4 Association between the core microbiome and leaf nutrients

The relationship between rhizosphere core microbiome and leaf micronutrients was analyzed using multiple linear regression models with leaf micronutrient concentrations (nitrogen, phosphorus, potassium, calcium, magnesium, iron, manganese, zinc, copper, boron, and sulfur) and the relative abundance of core microbiome in phylum, family, and genus levels. A *p* < 0.05 was considered to be significantly different, and 0.05 < *p* < 0.10 was considered as a tendency (Cha et al., 2023).

### 2.5 Co-occurrence network analysis

To predict bacteria–bacteria interactions in the gut microbial community, co-occurrence patterns of core bacterial families, and genera present in at least 50% of samples were evaluated in the network interface using pairwise Spearman’s rank correlations based on the relative bacterial abundance. The Spearman rank correlation was analyzed using *Hmisc* package (v. 5.2-2) within R Studio (version 1.1456). A significant rank correlation between two taxa (*r*_s_ > 0.5 or *r*_s_ < −0.5, FDR-adjusted *p*-value <0.05) was considered a co-occurrence event. The network was visualized using the Force Atlas algorithm in the interactive platform Gephi.^1^ In the network, nodes represent different taxa, and edges indicate correlations among nodes. The thickness of the edges indicates the strength of the correlation.

### 2.6 Statistical analysis

All statistical analyses were performed with GraphPad Prism (version 10.2.1). Leaf nutrients and gut microbiota were analyzed by t-test and the linear correlation between nutrients and soil microbiota was estimated by Pearson’s correlation analysis. Significant differences between treatments were defined at *p* < 0.05.

## 3 Results

### 3.1 The growth and nutrient levels vary between strawberry plants

To understand if rhizosphere microbiome composition is associated with plant growth and nutrient levels, we cultivated strawberry plants in the same soil under identical management practices. We classified the plants into healthy (H) and unhealthy (UH) groups based on morphological differences, particularly leaf color and shape (Figure 1). Strawberries in the H group exhibited dark green leaves and typical fruit shapes (Figure 1A), whereas UH plants had light green or brown leaves and irregularly shaped fruits (Figure 1B).

We measured the leaf concentrations of macro- and micronutrients from the randomly selected plants to understand differences related to growth status (Table 1). The nitrogen concentration was significantly higher (*p* = 0.006) in the leaves of strawberry plants in the H group compared to the UH group. However, the concentrations of other macronutrients, including phosphorus, potassium, calcium, and magnesium did not differ significantly. In contrast, the levels of iron (*p* = 0.050), manganese (*p* = 0.011), zinc (*p* = 0.009), and copper (*p* = 0.004) were significantly higher in strawberry plants of the UH group than in the H group. In conclusion, there was considerable variation in plant growth and leaf nutrient concentrations, although plants were grown under the same conditions.

**Table 1:** Nutrient concentrations in strawberries based on growth status.

	Healthy	Unhealthy	*p*-value
Macronutrients, %			
Nitrogen	2.82 ± 0.06	2.35 ± 0.14	0.004
Phosphorus	0.27 ± 0.02	0.25 ± 0.02	0.235
Potassium	1.79 ± 0.11	1.57 ± 0.15	0.137
Calcium	1.73 ± 0.14	1.99 ± 0.21	0.236
Magnesium	0.38 ± 0.03	0.37 ± 0.04	0.674
Micronutrients, mg kg^−1^			
Iron	190.43 ± 12.40	226.77 ± 14.45	0.050
Manganese	164.49 ± 18.80	233.35 ± 16.23	0.011
Zinc	76.80 ± 8.07	130.33 ± 12.97	0.009
Copper	21.13 ± 2.10	28.02 ± 1.75	0.004
Boron	78.09 ± 4.86	80.21 ± 7.26	0.733
Sulfur	1670.60 ± 38.81	1554.34 ± 73.38	0.056

### 3.2 Soil microbiome related to the growth status of strawberries

**Figure 2:**
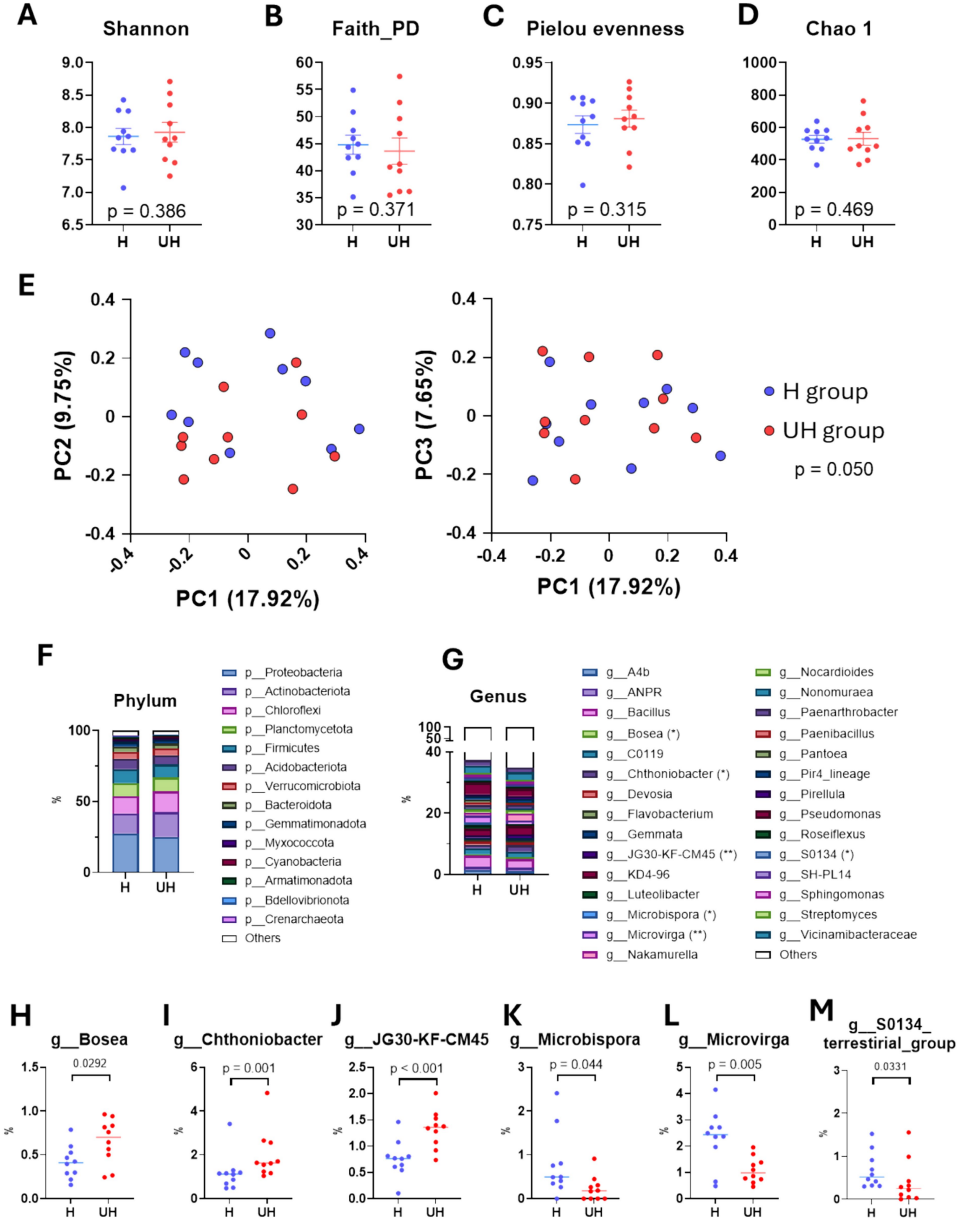
Rhizosphere microbiome of strawberry plants based on growth status. **(A–D)** Dot plot showing Shannon, Faith PD, Pielou evenness, and Chao1 index results. **(E)** PCoA plots of Bray–Curtis distances comparing soil microbiome between treatments (left; PC1 vs. PC2, right; PC1 vs. PC3). **(F)** Bar plot showing microbial abundance at the phylum level. **(G)** Bar plot showing microbial abundance at the genus level. **(H–M)** Dot plots showing significantly different genera (*Chthoniobacter*, JG30-KF-CM45, *Microbispora*, and *Microvirga*) according to the treatment groups.

To determine whether differences in growth and nutrient levels between the two types of strawberry leaves were associated with the rhizosphere microbiome, we analyzed soil microbiota. Their *α*-diversity indexes, including Shannon index (*p* = 0.386, measuring both species diversity and richness), Faith phylogenetic diversity (*p* = 0.371, reflecting the diversity of species), Pielou’s evenness (*p* = 0.315, assessing the uniformity of species distribution), and Chao1 index (*p* = 0.469, estimating species richness), were similar between the two groups (Figures 2A–D). However, microbiota structure differed significantly based on Bray–Curtis distances (*p* = 0.050; Figure 2E). The relative abundance of the phyla, Proteobacteria (27.64% vs. 25.11%), Actinobacteriota (13.95% vs. 17.53%), Chloroflexi (12.21% vs. 14.53%), Planctomycetota (9.24% vs. 9.86%), and Firmicutes (9.84% vs. 9.17%) were similar (Figure 2F). However, the relative abundance of specific genera varied according to growth performance (Figure 2G). The relative abundance of *Bosea* (*p* = 0.029), *Chthoniobacter* (*p* = 0.001), and JG30-KF-CM45 (*p* < 0.001) were significantly more abundant in the rhizosphere of UH group compared to H group (Figures 2H–J). In contrast, the relative abundance of *Microbispora* (*p* = 0.044), *Microvirga* (*p* = 0.005), and S0134 terrestrial group (*p* = 0.033) were significantly more abundant in the H group strawberries (Figures 2K–M). These findings collectively indicate that the rhizosphere microbiota associated with strawberry plants may contribute to regulating plant growth and nutrient levels.

### 3.3 Correlation analysis between leaf nutrient and soil microbiome

**Figure 3:**
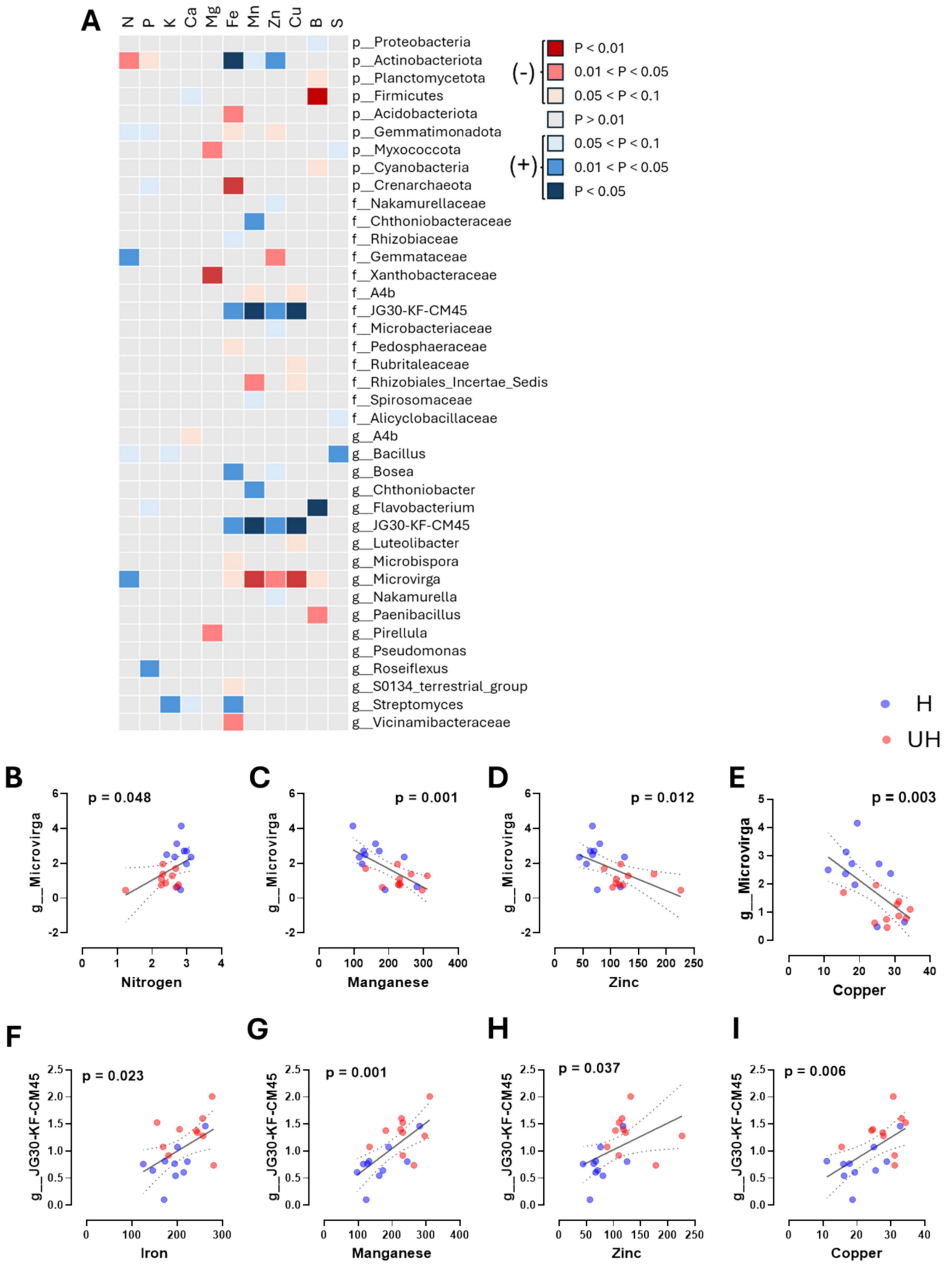
The relationship between leaf nutrients and core bacteria analyzed using linear correlation analysis. **(A)** Correlation between leaf nutrients (nitrogen, phosphorus, potassium, calcium, magnesium, iron, manganese, zinc, copper, boron, and sulfur) and the abundances of core bacteria analyzed using multiple linear regression models. Colors indicate significance levels, with red and blue representing negative and positive correlations, respectively. **(B–E)** Correlation between *Microvirga* abundance and leaf nutrient concentrations (sodium, manganese, zinc, and copper). **(F–I)** Correlation between JG30-KF-CM45 abundance and leaf nutrient concentrations (iron, manganese, zinc, and copper).

As we observed significant differences in the microbial communities within the rhizosphere of strawberries based on their growth status, we sought to determine whether the rhizosphere microbiome was associated with strawberry nutrient levels. To investigate this, we analyzed associations between key bacterial taxa and plant macro- and micronutrient levels using multiple linear regressions. Consequently, we identified three key bacterial taxa, phylum Actinobacteriota and genera *Microvirga* and JG30-KF-CM45 that were associated with plant nutrition levels. The relative abundance of *Actinobacteriota* was negatively correlated with nitrogen concentration (*p* = 0.031) but positively correlated with iron (*p* = 0.006) and zinc (*p* = 0.015) levels (Figure 3A). Similarly, JG30-KF-CM45 showed a positive correlation with micronutrient concentrations, including iron (*p* = 0.023), manganese (*p* = 0.001), zinc (*p* = 0.037), and copper (*p* = 0.006) (Figures 3B–E). In contrast, *Microvirga* showed an opposite pattern to the other two key bacteria, showing a positive relationship with nitrogen (*p* = 0.048) but negative correlations with manganese (*p* = 0.001), zinc (*p* = 0.012), and copper (*p* = 0.003) (Figures 3F–I). Notably, *Microvirga* in the well-grown group and JG30-KF-CM45 in the poorly grown group were identified as key bacteria. Their consistent correlations suggest that rhizosphere bacteria may be associated with plant nutrition levels.

### 3.4 Co-occurrence of microbial networks based on the growth status of strawberries

To elucidate the role of microbe–microbe interactions in shaping microbial community structure and function, we performed a co-occurrence network analysis. In the network, nodes represent distinct bacterial genera, while edges denote significant interactions, with blue edges indicating co-occurrence and red edges indicating co-exclusion relationships. Node size is proportional to the number of connections between each genus, highlighting dominant taxa within the community. Additionally, the analysis revealed modular structures within the network, where nodes sharing the same color represent taxa grouped into the same module. These modules indicate that bacterial groups that potentially co-exist within shared ecological or functional niches.

**Figure 4:**
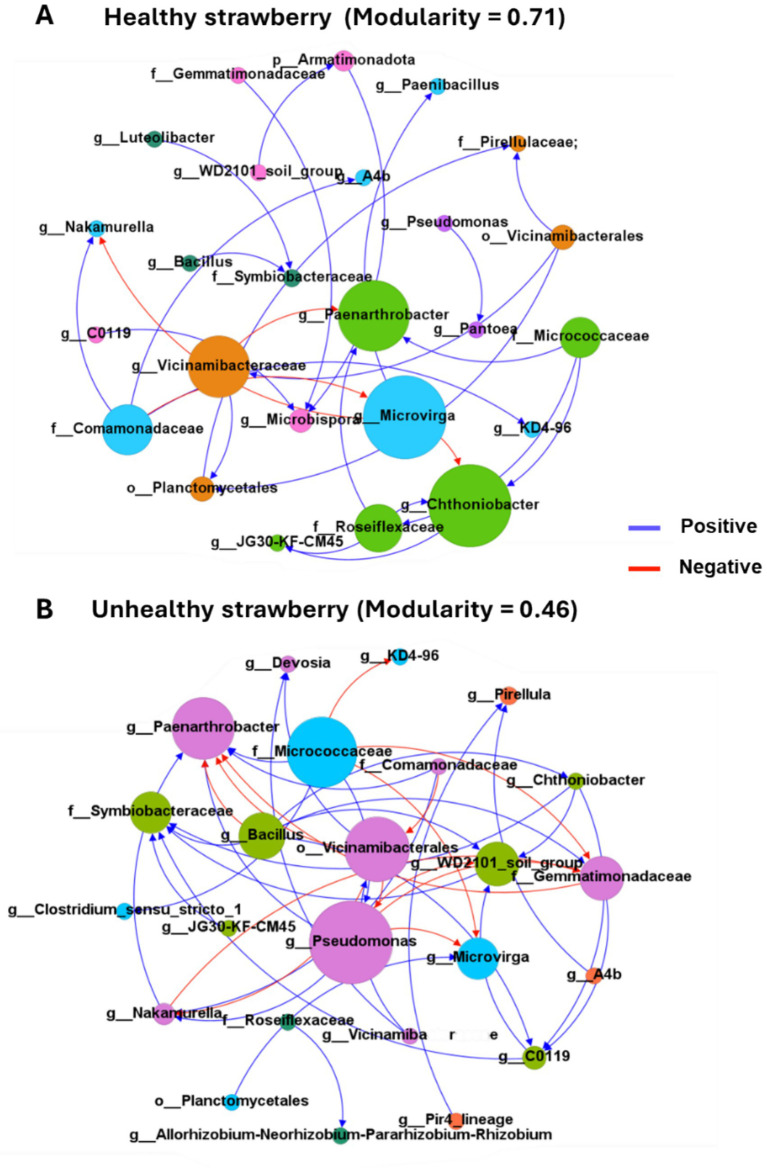
Co-occurrence of bacterial networks based on the growth status of strawberries (modularity value: 0.71 vs. 0.46). Co-occurrence networks predicting the bacteria-bacteria interactions among core bacterial genera in the H group **(A)** and the UN group **(B)**. Connections were detected using Spearman’s rank correlations (*r*_s_ > 0.5 or *r*_s_ < −0.5, FDR-adjusted *p* < 0.05). Dot sizes represent the number of connections per genus, and dot colors represent bacterial community clusters within the same module. Edge color represents either positive or negative associations between taxa.

In the microbial co-occurrence network of the H group, three primary modules were identified. The blue module dominated by *Microvirga*, the orange module dominated by *Vicinamibacteraceae*, and the green module dominated by *Paenarthrobacter* and *Chthoniobacter*. These taxa functioned as key hubs within their respective modules. Strong positive associations were observed between the blue and green modules, suggesting cooperative interactions that may contribute to community stability. In contrast, the orange module displayed competitive correlations with other modules, indicating potential antagonistic interactions that influence microbial community structure (Figure 4A). In the UH group, the purple module emerged as the dominant module, with *Paenarthrobacter* and *Pseudomonas* as hub taxa. This module exhibited strong negative correlations with other nodes, indicating a high degree of competitive interactions within the microbial network. Such competitive dynamics may account for the lower *M*-value (0.46) observed in the UH group compared to the H group (0.71), implying reduced community stability in the UH group (Figure 4B). Collectively, these findings underscore the pivotal role of bacterial interactions in modulating rhizosphere microbial community stability, thereby resulting in alterations in plant–microbiome interactions, even within the same soil environment. These changes can significantly impact plant health by modifying the nutrient dynamics within the rhizosphere.

## 4 Discussion

This study demonstrates that the rhizosphere microbiome is strongly associated with plant growth and nutrient uptake. Significant differences in microbial community structure were observed between the H and UH plant groups, with specific taxa, including *Microvirga* and *JG30-KF-CM45*, playing key roles in shaping microbiome composition. Moreover, specific taxa exhibited correlations with leaf nutrient concentrations, suggesting a potential role of rhizosphere-associated microbes in facilitating nutrient uptake and bioavailability. These findings underscore the critical importance of microbial interactions in maintaining plant health and productivity.

Significant differences in soil microbiota composition were observed based on the health status of strawberry plants (Figure 2). This microbial dysbiosis within the soil may disrupt essential physiological processes, thereby impairing the optimal growth and development of strawberries. Our results indicate that *Microvirga* is positively associated with nitrogen concentrations, suggesting its involvement in nitrogen metabolism within strawberry roots. The genus *Microvirga* comprises 17 validated species isolated from diverse environments, including human, aquatic, and terrestrial ecosystems. Notably, *Microvirga* is known for its ability to produce pigments and amylolytic enzymes via arsenic oxidation (Kanso and Patel, 2003; Caputo et al., 2016; Zhang et al., 2019). Han et al. (2024) reported a positive correlation between *Microvirga* and metabolites such as 0-oxononanoic acid, poly-D-glutamate, arginine, and calcium-phosphate (Ca–P), suggesting a pivotal role in carbon metabolism through nitrogen fixation. This metabolic function may contribute to plant growth promotion and enhanced resistance to environmental stress (Wang et al., 2022). Additionally, co-occurrence network analysis revealed significant interactions between *Microvirga* and other bacterial taxa in both H and UH strawberry rhizospheres. In soils from H strawberry plants, *Microvirga* exhibited a positive correlation with *Paenibacillus*, a known plant growth-promoting bacterium recognized for auxin production (Singh et al., 2024). In contrast, *Microvirga* was negatively correlated with *Pseudomonas* and *Micrococcaceae* in the rhizosphere of UH strawberry plants. In accordance with this study, Deng et al. (2019) also found that plant growth-promoting microbial modifications significantly altered the rhizosphere microbial community structure of strawberries, and the changes in the endogenous microbial community are closely related to plant nutritional status and health even under uniform environmental conditions. Because healthy plants are known to actively develop rhizosphere microbial communities, the enrichment of beneficial bacteria such as *Microvirga* in H plants may be partially explained by their introduction via root exudates. Conversely, abiotic stress or nutrient dysregulation in UH plants can disrupt these selection pressures, resulting in microbial imbalances characterized by increased competition and loss of functional redundancy. Collectively, these findings highlight the critical role of *Microvirga* in promoting plant growth not only through nitrogen fixation but also by fostering beneficial microbial interactions within the rhizosphere microbiome.

Optimal nutrient management is essential for successful strawberry cultivation, as a balanced supply of macro- and micronutrients directly influences plant growth, yield, and fruit quality (Tagliavini et al., 2005). Osvalde et al. (2023) established baseline standards for optimal nutrient concentrations in commercially grown strawberry leaves during the period 2014–2022. Our findings confirm that the UH group exceeded these optimal concentrations especially in micronutrients (iron, manganese, and zinc). Ruano et al. (1988) demonstrated that excessive zinc levels in strawberries result in growth inhibition, yield reduction, and impaired translocation of photoassimilates from leaves to roots. In addition, excessive zinc accumulation disrupts photosynthesis by reducing pigment levels, damaging photosystem II, and increasing reactive oxygen species (Paunov et al., 2018). Similarly, copper toxicity negatively impacts photosynthesis, nutrient uptake, and oxidative stress management, leading to chlorosis and growth impairment (Mir et al., 2021). Chlorosis, characterized by yellowing of leaves, results from disrupted chlorophyll biosynthesis or degradation of existing chlorophyll. Furthermore, excess copper induces reactive oxygen species production, causing oxidative damage to the chloroplasts and ultimately leading to necrosis of leaf tissues, which compromises plant health and productivity (Kaleem et al., 2024). Consistently, we observed yellowing of strawberry leaves, which may be attributed to the downregulation of photosynthesis caused by micronutrient dysbiosis.

The co-occurrence network, constructed using Spearman’s rank correlation analysis, is recognized as a powerful tool for elucidating the non-random distribution patterns of microbial communities (Barberán et al., 2012). In this study, co-occurrence network analysis of the rhizosphere microbiome from UH strawberry plants revealed a higher proportion of negative interactions compared to H plants, indicating intensified competition among bacterial communities. Such negative interactions are known to destabilize microbial communities, ultimately impairing plant growth (Lee et al., 2022). The UH group exhibited a comparatively higher number of positive interactions, with the microbial interaction network displaying an increased number of nodes and edges relative to the healthy group. Consistent with these observations, Zhang et al. (2023) reported that lower modularity is associated with reduced structural and functional stability in microbial communities. The low modularity observed in the UH group reflects a decrease in ecological compartmentalization, suggesting a breakdown in ecological niche partitioning and functional redundancy. This shift from cooperative to competitive microbial interactions may lead to reduced nutrient exchange efficiency and reduced plant resilience to environmental stress. Their study on microbial networks in diseased strawberry soils similarly revealed a higher number of nodes and edges, accompanied by lower modularity.

Although co-occurrence network analysis provides valuable insights into cooperative relationships between microbial taxa, it is important to note that such associations do not always imply direct interactions. Microbial members can influence host performance indirectly, even in the absence of direct contact (van der Heijden and Hartmann, 2016). In addition, despite the insights gained from this study, we acknowledge certain limitations. Specifically, the observational and correlative nature of the microbiome analyses does not allow for definitive conclusions regarding the causal role of *Microvirga* in promoting plant growth and shaping rhizosphere microbial communities. To address this limitation, future research should incorporate gnotobiotic systems or synthetic microbial community assays under controlled environmental conditions. These experimental approaches would enable precise functional validation of *Microvirga*’s contributions, both individually and in interaction with other microbial taxa, to plant physiological responses, nutrient assimilation, and microbiome assembly.

## 5 Conclusion

In conclusion, this study highlights the critical role of soil microbiome in regulating the growth, nutrient uptake, and overall health of strawberry plants. The findings emphasize the significant influence of microbial community composition, particularly the role of *Microvirga* and its associations with macro- and micronutrients, on plant development. Nutrient dysbiosis and altered microbial interactions, particularly in UH strawberry plants, were linked to impaired photosynthesis, chlorosis, and reduced plant productivity. Further studies to elucidate the specific mechanisms through which rhizosphere microbiota mediate nutrient cycling and plant-microbe interactions will provide insights into develop microbial-based strategies to enhance nutrient use efficiency and promote sustainable strawberry cultivation practices.

## Data Availability

The 16S rRNA gene amplicon sequencing data generated during the current study were submitted to NCBI under BioProject PRJNA1230227.

## Footnotes

^1^ http://gephi.org
